# Altered Metabolic Signaling and Potential Therapies in Polyglutamine Diseases

**DOI:** 10.3390/metabo14060320

**Published:** 2024-05-31

**Authors:** Alisha Vohra, Patrick Keefe, Prasanth Puthanveetil

**Affiliations:** 1 Chicago College of Osteopathic Medicine, Midwestern University, Downers Grove, IL 60515, USA; alisha.vohra@midwestern.edu (A.V.); patrick.keefe@midwestern.edu (P.K.); 2 College of Graduate Studies, Department of Pharmacology, Midwestern University, Downers Grove, IL 60515, USA

**Keywords:** polyglutamine diseases, metabolic signaling, NAD+ regulation, therapeutics, neuronal health

## Abstract

Polyglutamine diseases comprise a cluster of genetic disorders involving neurodegeneration and movement disabilities. In polyglutamine diseases, the target proteins become aberrated due to polyglutamine repeat formation. These aberrant proteins form the root cause of associated complications. The metabolic regulation during polyglutamine diseases is not well studied and needs more attention. We have brought to light the significance of regulating glutamine metabolism during polyglutamine diseases, which could help in decreasing the neuronal damage associated with excess glutamate and nucleotide generation. Most polyglutamine diseases are accompanied by symptoms that occur due to excess glutamate and nucleotide accumulation. Along with a dysregulated glutamine metabolism, the Nicotinamide adenine dinucleotide (NAD+) levels drop down, and, under these conditions, NAD+ supplementation is the only achievable strategy. NAD+ is a major co-factor in the glutamine metabolic pathway, and it helps in maintaining neuronal homeostasis. Thus, strategies to decrease excess glutamate and nucleotide generation, as well as channelizing glutamine toward the generation of ATP and the maintenance of NAD+ homeostasis, could aid in neuronal health. Along with understanding the metabolic dysregulation that occurs during polyglutamine diseases, we have also focused on potential therapeutic strategies that could provide direct benefits or could restore metabolic homeostasis. Our review will shed light into unique metabolic causes and into ideal therapeutic strategies for treating complications associated with polyglutamine diseases.

## 1. Introduction

Polyglutamine (polyQ) diseases are a group of nine inherited neurodegenerative diseases caused by trinucleotide repeats [1,2,3,4,5]. In this spectrum of identified polyglutamine diseases, the repeated trinucleotides result in a greater number than normal of repeated copies of the CAG codon that codes for glutamine in DNA translation [1,2,3,4,5]. As such, numerous excess glutamines are formed, resulting in an expanded polyglutamine tract.

Polyglutamine repeats often lead to aggregation in the body, especially neurons, leading to dysfunction and the degeneration of different neuronal cell populations [1,2,3,4,5]. Some of the dysfunctions include DNA damage, intracellular aggresomes, transcriptional dysregulation, and/or proteasomal degradation [1,2,3,4,5]. Furthermore, polyglutamine diseases can be inherited through transfer over generations or acquired with age throughout one’s lifetime. Recent research has concluded that the CAG repeat length is correlated with the onset of disease, with individuals with longer polyglutamine chains typically presenting with an earlier onset of polyQ diseases [1,2,3,4,5].

There are currently nine well-characterized polyglutamine diseases known to the medical community: Dentatorubropallidoluysian atrophy (DRPLA); Huntington’s Disease (HD); Spinal and Bulbar Muscular Atrophy (SBMA) or Kennedy’s Disease; Spinocerebellar Ataxia Type 1 (SCA1); Spinocerebellar Ataxia Type 2 (SCA2); Spinocerebellar Ataxia Type 3 (SCA3) or Machado–Joseph Disease (MJD); Spinocerebellar Ataxia Type 6 (SCA6); Spinocerebellar Ataxia Type 7 (SCA7); and Spinocerebellar Ataxia Type 17 (SCA17) [1,2,3,4,5].

This review focuses on the current literature, research, and understanding about the development, progression, and treatment of the group of polyglutamine diseases from a metabolic perspective. While others have looked at these considerations from more generalized, neurological, or clinical standpoints, we investigate here the metabolic components of polyglutamine disorders that are crucial to adequately comprehending the biochemical and cellular processes that the disease process must undergo to enact its changes and cause the clinical symptoms seen in patients.

## 2. Polyglutamine Diseases and Their Causes

Polyglutamine diseases are classified as those caused by a larger than normal number of glutamine repeats, which are encoded by CAG codons, in their respective gene. These repeats cause disease-specific clinical manifestations [1,2,3,4,5]. Each polyglutamine disease, its genetic defect, and its associated pathophysiology will be discussed below.

Dentatorubropallidoluysian atrophy (DRPLA) is caused by an expansion of CAG codons in exon 5 of the *ATN1* gene [6]. This gene codes for the atrophin 1 protein, also known as the DRPLA protein, which is a transcriptional co-regulator [7] that is commonly located in the neurons of the central nervous system [8]. Individuals with DRPLA have 49 or more glutamine repeats compared to the 7–34 that is typical of non-affected individuals, and this results in the jeopardization of neuronal cell survival [9]. DRPLA is known to have three major subtypes, each distinguished by the age of onset. Juvenile onset, which typically occurs before the age of 20 years old, often presents with symptoms of ataxia and progressive myoclonus epilepsy. Early adult onset, occurring between the ages of 20 and 40 years old, usually results in repeated seizures and potential myoclonus. Late adult onset, i.e., any onset after the age of 40, often presents with symptoms of ataxia, choreoathetosis, and dementia [10].

Huntington’s Disease (HD) is caused by an expansion of CAG codons within exon 1 of the huntingtin (*HTT*) gene [11,12]. This gene codes for the huntingtin protein, and an excess of polyglutamine repeats causes gene modifications and induces an incomplete splicing of the *HTT* gene [2]. Non-affected individuals have between 6–35 glutamine repeats in their *HTT* gene, while those with HD are known to have anywhere between 40–250 repeats. Clinically, those who present with Huntington’s Disease typically have progressive chorea, as well as numerous cognitive and behavioral symptoms [11,12]. 

Spinal and Bulbar Muscular Atrophy (SBMA), also known as Kennedy’s Disease, is the only X-linked polyQ disease. This recessive, X-linked condition is caused by an expansion of CAG codons in exon 1 of the androgen receptor (*AR*) gene, and it results in a loss of motor neurons in the spinal cord and brainstem [6]. This abnormal regulation of gene expression after ligand binding to disease accelerates disease progression [13]. SBMA has an adult onset, typically between the ages of 30 and 60 [14]. Clinically speaking, SBMA causes widespread weakness along with atrophy and fasciculations related to the degeneration of lower motor neurons in the spinal cord and brainstem [15] and postural tremors and muscle cramps, which are followed by progressive muscle weakness ([14,16,17]). The distinguishing features of SBMA include profound facial fasciculations, bulbar signs, gynecomastia, as well as sensory disturbances [6]. Since the polyglutamine repeat expansion occurs in the androgen receptor gene, males are more susceptible to the SBMA disease [18]. 

Spinocerebellar ataxias [19] vary in type, and each targets a specific gene and thus presents with differing clinical symptoms. All ataxias, except for Spinocerebellar Ataxia Type 6 (SCA6), undergo somatic expansion. 

Spinocerebellar Ataxia Type 1 (SCA1) is caused by an expansion of CAG codons within the exons of the *ATXN1* gene, which codes for ataxin-1 protein [6]. This protein is involved in transcriptional regulation and also RNA metabolism [20,21]. Onset is usually in the mid-30s, and SCA1 is known to progress much faster than the other SCAs [22]. Patients with SCA1 experience progressive ataxia in addition to impaired cognition, the weakening of eye muscles, dysarthria, and dysphagia, among other symptoms [23,24].

Spinocerebellar Ataxia Type 2 (SCA2) is caused by an expansion of the CAG codons within the exons of the *ATXN2* gene, which produces the ataxin-2 [6] protein. This is a cytoplasmic protein that is most commonly found in Purkinje fibers [25]. The aggregation of ataxin-2 has been known to cause SCA2 in both humans and mice. In this condition, the excess glutamine-containing mutant form of ataxin-2 causes Purkinje cells to undergo glutamate-induced apoptosis [26]. Individuals with SCA2 can experience the onset of the disease between the ages of 2 and 65, although it will usually present more aggressively if onset is before the age of 20 [23,24]. Clinical presentation includes progressive ataxia, dysarthria, postural tremors, slow saccades, and hyporeflexia, among other symptoms [27].

Spinocerebellar Ataxia Type 3 (SCA3), also known as Machado–Joseph Disease (MJD), is caused by an expansion of the CAG codons in the exons of the *ATXN3* gene, which produces ataxin-3 [6]. This gene interaction is thought to activate the apoptosis that induces ataxia [28,29]. As a protein, ataxin-3 interacts with Rad23, a UV excision repair protein and valosin-containing protein (VCP). Together, ataxin-3, Rad23, and VCP create a protein complex that assists with protein degradation in the cell [30]. Another possible mechanism that could lead to disease pathogenesis is the excess polyglutamine expansion resulting in the misfolding of proteins that ultimately induces ubiquitination through the activation of Parkin, an E3 ubiquitin ligase [31]. Parkin is known to promote ubiquitination and the degradation of ataxin-3 [32]. Onset of SCA3 can be anywhere between the ages of 5 and 75. In terms of clinical manifestation, patients with SCA3 experience progressive cerebellar ataxia, areflexia, spasticity, and muscle atrophy, among other symptoms [33,34].

Spinocerebellar Ataxia Type 6 (SCA6) is caused by an expansion of the CAG codons in exon 47 of the 3′ region of the *CACNA1A* gene, which codes for brain-specific, voltage-dependent calcium channels that are often found in Purkinje cells [35]. It is the only spinocerebellar ataxia that does not somatically expand. Patients with SCA6 tend to experience progressive ataxia, dysarthria, dysphagia, intention tremors, along with positional vertigo, nystagmus, and other motor and muscular defects [27]. Onset can be anywhere between 19–73, with the average age being between 43–52 years old [36]. 

Spinocerebellar Ataxia Type 7 (SCA7) is caused by a CAG expansion within the *ATXN7* gene that results in an unstable polyglutamine chain within the ataxin-7 protein. This surplus of glutamine in the ataxin-7 protein, which is commonly found in the cytoplasm and nucleus of nerve cells [37], can cause the degeneration of rods, photoreceptors, as well as Purkinje cells [38]. Ataxin-7 also acts to control multiple transcriptional coactivator complexes that can acetylate histone H3 [39,40,41]. Most often, the onset of SCA7 occurs during the childhood years, and its clinical features include macular and/or retinal degeneration with vision loss, slow saccades, ophthalmoplegia, as well as progressive ataxia and dysphagia [42]. 

The last of the known polyglutamine diseases is Spinocerebellar Ataxia Type 17 (SCA17), which is caused by CAG or CAA repeat expansions of 45 or more within the *TBP* gene [43]. TBP is a DNA binding subunit of the RNA polymerase II transcription factor D (TFIID) and is important for protein-encoding gene expression [44]. The onset of SCA17 is usually between ages 3 and 55, with the full penetrance of symptoms being experienced around 50 years of age in patients [45,46]. The clinical features of SCA17 include progressive gait; limb ataxia; seizures; neurologic, cognitive and/or psychiatric impairments; as well as pyramidal and extrapyramidal features such as spasticity, chorea, and dystonia [47]. The list of polyglutamine diseases, with the genes involved and major clinical features, are described in Table 1.

### Demographics and Available Treatment Options

Study reports from small cohorts in European populations have demonstrated that SCA1 disease prevalence is around 0.2–2/100,000 subjects [6,51]. The frequency of occurrence being 4% in males and 6% in females, with the mean age for the onset of disease being 38 years. The SCA2 prevalence is around 0.1–5.8/100,000 subjects, with the prevalence being split at 6% in males and 2% in females. The mean age of onset for SCA2 is 36 years [6,51,52,53,54]. For SCA3, the prevalence is 0.6–0.8/100,000, with 1% frequency in males and females and the average age of onset being 40 years [6,51,52,53,54]. For SCA 6 and SCA 7, the prevalence is 0.5 and 0.2 per 100,000, and the average onset of age is 53 and 32, respectively [51,52,53,54]. The prevalence for SBMA and DRPLA is 0.3 and 9.5 per 100,000, with the average age of onset being at 45 and 32 years, respectively [51,52,53,54]. HD has the maximum prevalence of 10.6–13.7 per 100,000, with a frequency of occurrence of 13% in males and 6% in females and the average age of onset being 40 years [6,51,52,53,54]. Regarding the treatment options, agents like Buspirone—which is known to have anxiolytic effect due to the dopaminergic receptor, D2—and the 5-HT-1A activator have been tested for their ability to normalize gait in SCA1, 2, 3, and 6 [6]. Similarly, valproic acid is a known anti-epileptic, it is known to act by inhibiting the voltage-gated sodium channel, and it has been used in attempts to reduce tremor, especially in SCA3. Riluzole, an inhibitory agent for glutamate release and also known to inhibit voltage-gated sodium channels has been shown to have a neuroprotective effect by decreasing excitotoxicity for all major forms of ataxia, including SCA1 and 2 [6,55]. Agents like amantadine, which is an N-methyl-D-aspartate activator, have been used in clinical studies to control uncontrollable involuntary movements or tremors for SCA7 and HD, and they have shown some promise [52,53,54]. Traditionally known as a diuretic, acetazolamide, which is known to inhibit the carbonic anhydrase enzyme, has shown promising pharmacological benefits in controlling symptoms like unwanted eye movements and the vertigo mostly seen in SCA6 [55,56,57]. Varenicline, a partial agonist for the nicotinic acid receptor, has demonstrated some level of support against cerebellar symptoms like uncoordinated movement and speech, eye movements, and the vertigo that accompanies SCA3 disease [55,56,57]. Agents like tetrabenazine and deutetrabenazine, which are agents known to inhibit mono amino re-uptake by binding to its uptake receptor (called vesicular monoamine transporter type 2 (VMAT2)), have been shown to prevent involuntary movements in HTT [55,56,57]. Olanzapine, which is a known inhibitor of central dopaminergic and 5-HT receptors, has shown added benefits in HTT by reducing disorganized speech and behavior. The thyrotropin-releasing hormone has shown promising effects on decreasing ataxia symptoms for all SCA phenotypes [55,56,57]. Nusinersen, which is an antisense oligonucleotide-based therapy intended to increase Survival Motor Neuron (SMN) proteins, and risdiplam, a chemical agent that is known to modify splicing and to enhance the gene and protein levels of SMN, are known approved agents for SBMA (Kennedy’s disease) [58,59,60]. Yet, all the agents previously mentioned come with either systemic adverse effects or limited abilities in controlling only the symptoms. Understanding the metabolic dysregulation during these disease conditions could give us insights in how to strategize new therapies, which could provide additive pharmacological effects with the existing therapeutic agents.

## 3. Common Metabolic Dysfunctions in Polyglutamine Diseases

Metabolic dysfunctions are inexplicably intertwined with polyglutamine disease development and progression. While it is known that polyglutamine diseases, at their core, are caused by an excess of glutamine repeats in a subset of genes, it is now clear that the downstream effects of such excesses result in several metabolic shifts in cells. Major changes include mitochondrial metabolism modifications, DNA damage, and subsequent protein misfolding. Such changes result in the need for response and repair mechanisms and the induction of cellular apoptosis, which often cause polyglutamine disease symptoms, just to name a few. However, the specifics of such changes are not always limited to the group of polyglutamine diseases, they can be unique to the disease itself. 

The development of Huntington’s Disease (HD) has been attributed to an increase in astrocyte dysfunction; however, the potential metabolic mechanisms within this association were unclear prior to Lange et al.’s work [61]. Lange and colleagues explored the connections between polyQ length and gene expression and the overall damage response within astrocytes to see if a significant relationship in either condition exists [61]. In induced pluripotent stem cells (iPSC) astrocyte lines, RNA sequencing, and metabolic analysis, higher polyQ length was revealed as being correlated to astrocyte reactivity and related metabolic changes. Furthermore, the aforementioned study concluded that Huntington Disease (HD) astrocytes did experience increased DNA damage, damage response, and an increased presence of mismatch repair genes and proteins; this was likely due to the multitude of transcriptional changes and the increase in polyQ length that occurred due to a larger than normal amount of CAG repeats [61]. 

### 3.1. Role of the Defective Mitochondrial Function

The metabolic dysfunctions related to the processes of the mitochondria are also thought to be causative and progressive in some, if not all, of the polyglutamine diseases. Many of the defining characteristics of SCA7 are those recognized as symptoms of mitochondrial disorders, which are known to result in the altered energy metabolism of the cell. Based on available published reports, it is believed that mitochondrial dysfunction could be a crucial component of SCA7 pathogenesis [62]. For example, SCA7 often manifests in patients with symptoms of inefficient oxygen consumption and issues with respiratory exchange, which are distinctly mitochondrial origin in nature [62]. Cerebellar Purkinje cells from SCA7 mice demonstrate mitochondrial network abnormalities, including an enlarged mitochondrial size. Further, in conditions with human stem cell knockouts, mitochondrial defects, impaired oxidative metabolism function, and reduced NAD+ production were found. The aforementioned study concluded that the mitochondrial dysfunctions that are due to a net reduction in NAD+ production are a defining pathogenic characteristic of SCA7 [62].

Aside from altered energy metabolism, mitochondrial-related apoptosis is thought to be another mitochondria-related metabolic dysfunction that is induced within polyglutamine and, to a larger extent, neurogenerative diseases. Operating through mechanisms of increased oxidative stress and corresponding redox imbalances, altered calcium metabolism, impaired bioenergetics, and/or abnormal trafficking, it is thought that shifts in any of these areas could result in mitochondrial dysfunctions that cause apoptosis of the cell [63]. With Huntington’s Disease in particular, HTT is known to increase Dynamin related protein 1 (Drp1), which is a crucial protein that regulates mitochondrial fission. Thus, an increase in Drp1 could assist in mitochondrial degradation, as well as in its corresponding degradation and dysfunction that leads to the both the increase in and vulnerability for the apoptosis that occurs within the cells of Huntington’s Disease patients [64].

Yeast models have been successful in determining the potential relationship between the development of the polyQ disease and the mitochondrial dysfunction that ultimately result in metabolic changes in cells [65]. Papsdorf et al. used yeast models and genomic analyses, microarrays, and NMR-based metabolic analysis to determine which genes are most likely to exhibit polyglutamine toxicity if deleted or mutated, to understand the transcriptional response if polyglutamine toxicity does indeed occur, and to finally see the mitochondrial metabolic response to these genetic changes [65]. The research team identified 14 unique genes whose deletions resulted in polyQ toxicity, with many of them coding for mitochondrial fusion proteins and indicating a direct relationship between the mitochondrial fusion and polyglutamine chain length [65]. Microarray analysis determined that, transcriptionally, there was an upregulation in the genes that code for components of sulfur and iron metabolism and, correspondingly, mitochondrial iron–sulfur cluster formation. These changes are known to result in an imbalance of iron concentration in the cell and an overall reduction in the activity of mitochondrial iron–sulfur clusters [65]. This is of particular importance because these clusters contain aconitase, a crucial enzyme used in the citric acid cycle and thus an important contributor toward cellular respiration and metabolism. Consequently, the researchers were able to conclude that mitochondrial metabolism is significantly reduced in the presence of polyglutamine toxicity, which causes an accumulation of metabolic intermediates, thus resulting in a protein aggregation that often presents as identifiable polyQ disease symptoms.

### 3.2. Role of Misfolded Proteins

Protein misfolding is another potential metabolic theory regarding the cause of polyglutamine disease development and progression. Nagai and colleagues hypothesized that the conformational change of polyglutamine chains from an alpha-helix to beta-sheet formation is correlated to cellular toxicity [66]. They thought that an increase in the number of polyQ repeats in a chain induce the beta-sheet conformation, which creates protein aggregations that manifest as polyglutamine diseases [66]. However, subsequent studies have not been able to confirm this correlation [67,68]. As such, the toxicity of expanded polyQ proteins is also thought to be due to aberrant protein interactions leading to aberrant functions [67]. 

Protein quality control systems (i.e., chaperones, ubiquitin ligases and proteasomes, and autophagy) have been investigated further for their role in the dysfunction in the polyQ disease. Chaperones, in particular, are known contributors to the alleviation of polyglutamine protein aggregation, either through a direct prevention of the aggregation or through modulation of the sequences that flank the polyQ stretch [69]. Researchers have concluded that the only known chaperones that can physically act upon the beta-sheets and beta-harpins that result in a dysfunction caused by an expanded polyglutamine tract are DNAJB6 or DNAJB8, which are both related to the heat shock protein 70 (HSP70) family. HSP70s are a group of chaperones commonly expressed in times of protein stress; when expressed, they act as suppressors of aggregation in many polyglutamine diseases [67,68]. The current theory regarding the aggregation mechanism is that it occurs in two stages. First, the non-polyQ-containing domains of the protein in question are nucleated. Once this occurs, aggregation in the polyQ stretch is sped up due to the impact of the formed nucleus acting as an anchor. This initiates a feedback loop that culminates in an aggregate [70]. While the specific means and players vary between the polyglutamine diseases, the opening of hydrophobic, aggregation-prone motif areas within the non-polyQ regions of the protein lead to the unwanted formation of the initial nucleus that starts the aggregation process. Such motifs are normally buried in the gene or are actively interacted with by binding partners, both of which can influence aggregation. The fragmentation hypothesis of aggregate and, thus, disease formation has been seen in DRPLA, SBMA, and some SCAs, although it is less proven for SCA1, SCA2, and SCA17. However, this hypothesis highlights the important role that proteases play as the inhibition and mutation of proteases give rise to increased disease progression. Therefore, proteases, along with chaperones and other post-translational modifications, represent areas that can, and should, be targeted for polyglutamine disease treatment [71].

Metabolism is heavily affected by the changes induced by polyglutamine disease formation. These shifts in energy metabolism, DNA damage and repair, and cellular apoptosis and homeostasis often result in the symptomatic manifestations of the polyglutamine disease that clinicians see. Thus, these metabolic pathways and quality control mechanisms at hand can be helpful in the future as new and more specific therapeutics are developed.

## 4. NAD+ Regulation in Polyglutamine Disease and Therapeutics

Nicotinamide adenine dinucleotide (NAD+) is a coenzyme used in many redox reactions, especially those involved in energy metabolism. NAD+ can be made cellularly from L-tryptophan through the kynurenine pathway or via vitamin precursors through the Preiss–Handler pathway. In addition to its role as a coenzyme, NAD+ also serves as a co-factor for sirtuins (such as Sirt1), CD38, CD157, and poly(ADP-ribose) polymerases (PARPs), which all consume NAD+ as part of their roles in metabolic regulation, immune response, DNA repair, autophagy, and aging [72].

Previous research that has detailed the potential role of mitochondrial dysfunction underlying the metabolic disturbances causing polyglutamine diseases has indicated that a reduction in NAD+ production may be at the root of polyglutamine disease formation and progression. When tested in human stem cell knockout conditions, researchers have found numerous mitochondrial defects and impaired oxidative metabolism in the cells with expanded polyQ chains specific to SCA7. Since NAD+ holds a crucial role in metabolic regulation that would likely affect mitochondria and oxidative phosphorylation, it was concluded that the reduction in NAD+ is a likely culprit of SCA7 pathogenesis [62].

One of NAD+s main roles involves maintaining the normal aging processes through metabolic regulation. There is a known, age-dependent reduction in NAD+ that serves as a part of what is considered to be the normal aging. While the underlying reason for this reduction is still unknown, it is thought that it could be related to age-related brain deterioration (as NAD+ is needed in adequate amounts for proper brain function) due to the fact that a reduction in NAD+ production occurs with age or that there is increased consumption of NAD+ by CD38-CD157 and/or PARPs for their roles in metabolism. With regard to Huntington’s Disease in particular, researchers have determined that there are impairments within the kynurenine pathway that result in the reduction in NAD+ production that is characteristic of Huntington’s Disease and presumably other polyQ diseases. Furthermore, Huntington Disease patients have been known to have less than normal levels of kynurenic acid and larger than normal levels of 3-hydroxykynurenine and quinolinic acid, all of which end up in an accumulation of kynurenine intermediates and an overall reduction in NAD+ production. As such, it is thought that these changes cause elevated intracellular calcium levels, impaired mitochondrial functions, and elevated glutamatergic neurotransmission in neurons, which ultimately result in neuronal dysfunction and induce cellular death through autophagy [62]. Thus, the role of NAD+ precursors and the kynurenine pathway may serve as possible therapeutic targets for the treatment of polyglutamine diseases in the future [73].

**Figure 1:**
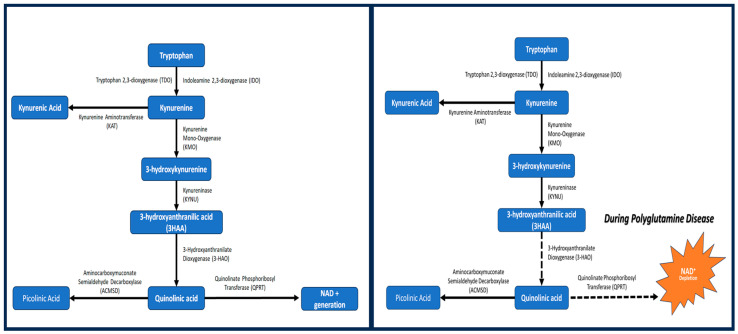
Altered NAD+ regulation during polyglutamine diseases. Tryptophan metabolism is responsible for the generation of NAD+. Kynurenine needs to undergo further metabolism to generate quinolinic acid with a subsequent generation of NAD+. During polyglutamine disease, the tryptophan metabolism could be altered, leading to an enhanced kynurenic acid generation and decreased formation of quinolinic acid with a resultant decrease in NAD+ generation.

Sirtuins are a group of NAD+-dependent acetylases that act as signaling proteins within metabolic processes. Recent reports have indicated that this group of acetylases may serve as a future therapeutic target to slow down cellular aging [74]. Sirtuin 1 (Sirt1) is a particular sirtuin known to be capable of reversing age-related neurodegeneration. Stoyas and colleagues looked into SCA7 patients and discovered that those with this polyglutamine disease have downregulated calcium flux genes that manifest, particularly with the activation of the potassium channels in Purkinje cells, as abnormal calcium-dependent cerebellar membrane excitability [75]. The degeneration of cerebellar Purkinje cells has been previously associated with causing inherited ataxias due to the dysfunction of the inositol (1,4,5) triphosphate receptor signaling pathway, which regulates the calcium release from the endoplasmic reticulum of cells. These alterations in the phosphatidyl-inositol and calcium homeostasis pathways were localized to the cerebellum of those with SCA7, and they were accompanied with a reduction in Sirt1 activity, as well as PARP-1 upregulation. The researchers used these findings to investigate mice with SCA7 and found that Sirt1 overexpression resulted in the “rescue” of neurodegeneration and calcium flux defects, as well as NAD+ repletion. Additional explorations into Sirt1 have found that increased expression can help relieve Huntington’s Disease phenotypes [76]. Furthermore, SCA7 mice have been found to have a predisposition to DNA damage and a preference toward the PARP-1 utilization of NAD+ stores in the cell. The vulnerability to DNA damage causes the SCA7 symptoms through mutations in the DNA repair protein genes, and cerebellar Purkinje cells are particularly affected due to the fact that they are very metabolically active and require more regular DNA repair [75]. Overall, PARP-1 overactivation directly induces NAD+ depletion, resulting in a decrease in Sirt1 expression, which is seen across the board in SCA7 patients [75]. While it is known that Sirt1 does indeed play a crucial role in NAD+ regulation, the actual mechanism by which Sirt1 contributes to NAD+ repletion is unclear. One prevailing theory is that it may be performed through the autophagy of cells with larger than normal polyglutamine tracts helping to rid the cell of their toxicity [77,78]. Recent pre-clinical studies in rodent models have demonstrated a drastic decrease in the NAD+ levels in SBMA (Kennedy’s Disease). When supplemented with the NAD+ precursor, nicotinamide riboside (NR) is not able to normalize NAD+ levels. This points to a lack of a proper pharmacological agent that could effectively enhance systemic NAD+ levels [79]. Recently, pharmaceutical research utilizing beta-lapachone (beta-lap), a naturally occurring quinone, has found that the substance induces autophagy by activating Sirt1, resulting in the reduction in polyQ aggregates and, thus, cellular toxicity. Sirt1 deacetylates a number of autophagy-related proteins (Atg5, Atg7, and Atg8), thus resulting in the initiation of autophagy [80]. Beta-lapachone, in particular, is relevant as it is a substrate of NADH:quinone oxidoreductase (NQO1); thus, it uses NADH as an electron source, produces NAD+, and utilizes NAD+ when re-oxidizing [81]. Changes in NAD+ levels result in consequential changes in the expression of Sirt1 in cells. As such, beta-lapachone and other quinones serve as another category of potential therapeutic targets for the treatment of polyglutamine diseases [82]. Overall, following polyglutamine diseases, the tryptophan metabolism could be in an imbalance, resulting in the depletion of NAD+ or decreased NAD+ generation (Figure 1). Given the current literature, NAD+ holds important roles in many metabolic processes, especially in those that regulate DNA repair, cellular death, and the aging of cells. The ability of Sirt1, a NAD+-dependent enzyme, in rescuing neurodegeneration demonstrates that NAD+ regulation is an ideal strategy for polyglutamine diseases therapeutics.

## 5. Glutamine Metabolism in Polyglutamine Disease and Therapeutics

Glutamine, due to the nature of disease formation, is at the crux of the progression and manifestation of polyglutamine disease. Most of its function in the body relates, in one way or another, to the glutamate–glutamine cycle (GGC), which allows for the conversion of glutamate (the primary excitatory neurotransmitter in the brain and central nervous system (CNS)) and glutamine (which is a substrate and used as an energy source). The glutamate–glutamine cycle is a critical type of regulation due to its ability to recycle and clear glutamate and thus eliminate excess excitatory signals and associated responses. When this cycle is altered, potentially through the overaccumulation of glutamine molecules, glutamate clearance often becomes compromised, leading to excess neuronal stimulation and thus a turning on of the cell death pathways. This could also be a prominent mechanism of neuronal toxicity and death during polyglutamine diseases [83,84,85].

In the CNS, glutamine is synthesized by astrocytes from glutamate using the enzyme glutamine synthetase [86] and ammonia. Once formed, the glutamine molecules are moved around by glutamine transporters that are able to transport glutamine out of the astrocytes and into the glia, neurons, and other cell types, along with other amino acids and ions, using both symport and antiport mechanisms [83,84,85]. A critical component of glutamine regulation is through the expression of glutamine synthetase. Research into one subtype, glutamine synthetase 1 (GS1), has found that the expression of GS1 helps to alleviate the mortality and pathologic defects present in *Drosophila* flies that present with mutant huntingtin, which is known to cause Huntington’s Disease. In a *Drosophila* model, Vernizzi and colleagues revealed that the presence of GS1 favors autophagy, which significantly reduces huntingtin aggregates [87]. Mechanistically, GS1 expression prevents the activation of the mTOR and downstream signaling pathway, including S6Kinase (a major downstream target of mTOR that is accompanied by a reduction in amino acid levels of arginine) and asparagine (which is known for mTOR activation). Changes in the amino acid levels through GS1 regulation can create “starvation-like” conditions that ultimately force autophagy to occur. GS1 protein levels have also been found to be reduced in the fibroblasts of Huntington’s Disease patients, indicating a connection between GS1 dysregulation and polyglutamine disease [87].

### 5.1. Role of Polyglutamine Tracts

Polyglutamine diseases have also been found to be unique in that the polyglutamine conformation results in stability as opposed to poly forms of other amino acids that are relatively unstable. Research into the structure of polyglutamine tracts has found that, while all L-polypeptides form into a µ-helix (which is a tubular and pore forming arrangement), poly-L-glutamine results in a stable structure that is unlike that of other amino acids, and it also acts as an ion channel when incorporated into a membrane. Researchers have brought their thoughts together into the “toxic channel hypothesis”, which states that, in cases of polyglutamine diseases, proteins that have a larger than normal number of glutamine repeats are proteolyzed to produce fragments that also have long polyglutamine stretches. These stretches form into the aforementioned ion channels when inserted into cellular membranes, are cation-selective particularly to monovalent cations, and have long open states. All of this induces cytotoxicity in the cell. During such states, the cation flow disrupts the potential on the cellular membranes and dissipates the proton gradients necessary for the normal metabolic functions in cells. In the mitochondria in particular, the dissipation of the proton gradient greatly affects energy formation and the metabolism processes, ultimately leading to cell death and more physical symptoms of polyglutamine diseases [88]. As such, the structure of polyglutamine tracts as molecules and its interactions with its cellular surroundings play important roles in the overall polyglutamine disease formation.

### 5.2. The Role of Glutamate and Glutamate Transporters

Aside from its role in producing glutamine and serving as the primary excitatory neurotransmitter of the central nervous system, glutamate has a number of other roles that contribute to metabolism and are of note with regard to polyglutamine diseases. Glutamate produces alpha-ketoglutarate, an intermediate of the citric acid cycle, through glutamate dehydrogenase (GDH). GDH along with aspartate aminotransferase (AAT) are the two main enzymes that mediate glutamate synthesis and metabolism in the brain. GDH is primarily found in mitochondria and AAT, and it works both in mitochondria and cytosol. Mechanistically, when glutamate enters the citric acid cycle, there is an increase in the amount of citric acid intermediates as the glutamate is converted into alpha-ketoglutarate via GDH, thus enhancing tricarboxylic acid cycle (TCA) intermediates [84].

Due to glutamate’s prevalence and importance in the central nervous system, glutamate transporters act as important regulators of the excitatory responses in the brain and body. The central nervous system houses two main types of transporters related to glutamate, including the sodium-dependent transporters and cysteine–glutamate exchangers. The former being a symporter that co-transports two or three sodium molecules and a proton with a single glutamate, while the latter of the two provides a source of cysteine to cells that are producing glutathione, which acts as an antioxidant in the body. One such transporter, GLT-1, is a presynaptic glutamate transporter involved in many of these response mechanisms. GLT-1 is the predominant transporter of the neuroaxis, and while it is not expressed in astrocytes, it is found very readily within brain tissue. In normal conditions, due to their prevalence, these transporters provide quite a large capacity for glutamate clearance. As such, future therapeutics that target them are needed in high inhibitory concentrations. However, any substantial decreases in transporter expression leads to neuropathology, with affected tissues becoming more vulnerable to excitotoxicity from the overexpression of glutamate and the excitatory response that it provides [89].

Brain GLT-1 levels have been found to be significantly reduced in patients with Huntington’s Disease. Along with this reduction in expression, the downregulation of *SLC1A3* and *SLC1A6* genes, which code for glutamate transporters GLAST and EAAT4, were noted. All of them indicated a clear role in glutamate uptake within Huntington’s Disease pathology and disease progression [84]. Researchers have also found that mutant huntingtin (mHtt) does not bind to the post-synaptic density protein 95 (PSD-95) as much as the non-mutant type. This leaves PSD-95 to bind instead to N-methyl-D-aspartate (NMDA) receptors (a type of glutamate receptor), which thus increases the activation of the receptors. The combination of increased NMDA receptor activation and stabilization, as well as decreased glutamate uptake, makes affected cells vulnerable to excitotoxic cell death, thus leading to pathology. Further, the aggregates of mHtt in glial cells have been correlated with a decrease in GLT-1 protein expression, showing that a reduction in glutamate transporters is a likely cause in the aggravation of Huntington’s Disease symptoms [89].

Glutamate transporters and glutamate uptake have also been related to a few spinocerebellar ataxias in addition to Huntington’s Disease. Custer et al. found that the polyQ expansion in SCA7 is specifically expressed in the Bergmann glia of the cerebellum [90]. Another extension of the role of glutamate transporters involves SCA5, which can be caused by a mutation in β-III spectrin and is expressed in Purkinje neurons. Under normal conditions, β-III spectrin helps stabilize EAAT4, another type of glutamate transporter, on the cell surface [91]. The EAAT4 protein expression is regulated in conjunction with calbindin, which is a Purkinje cell that is specific regulatory protein; as such, when it is reduced, glial dysfunction and the corresponding decrease in glutamate uptake can be added to the contributors of the development of SCA5 through the continued excitatory response of the excess glutamate [89].

### 5.3. Glutamate–GABA Balance as a Determining Factor

In contrast to the effects of glutamate, gamma-aminobutyric acid (GABA) acts as the primary inhibitory neurotransmitter in the central nervous system. Glutamate can be converted to GABA in both neurons and astrocytes by the glutamate decarboxylases GAD65 and GAD67. Once formed, GABA is put into vesicles and is transported via vesicular GABA transporters (VGAT), and it is released into a synapse after membrane depolarization occurs to elicit an inhibitory signal [86]. Additionally, astrocytes can take up GABA, turn it into α-ketoglutarate, and then turn it back into glutamate through the citric acid cycle, thus making GABA a precursor for TCA cycle intermediates. Current research shows that GABAergic dysfunction is present in many polyglutamine diseases; in particular, it is a dysfunction in the GABA signaling pathway in those with Huntington’s Disease [92]. Analysis of the GABA levels in patients with Huntington’s Disease has indicated that these patients have lower than usual levels of GABA, suggesting that there could be either a lower activity of the glutamate decarboxylases that produce GABA or an increase in GABA transporters (such as GAT1 or GAT3), which are enzymes used in GABA degradation (GABA-T) or GABA release channels in the glial cells (like Best1). Furthermore, it is thought that the mutant huntingtin protein aggregates could cause a suppression of the GABA receptors and their downstream components [93].

### 5.4. Glutathione Regulation in Polyglutamine Disease

Glutathione is another molecule that is of relevance within glutamate and glutamine metabolism. An antioxidant in the body, glutathione has roles in signaling, redox reactions, and oxidative stress management, as well as a protectant, particularly in the central nervous system. The molecule is synthesized in two steps, both of which require ATP for energy: in the first step, glutamate and cysteine are combined via glutamate-cysteine ligase (GCL) to form γ-glutamyl cysteine; in the second step, glycine is added by glutathione (GSH) synthase to form GSH. The current literature seems to show that both GSH levels and that of GSH-producing and GSH-dependent enzymes are likely abnormal, and that the enzymes are not fully functional in the presence of a polyglutamine disease (Huntington’s Disease more specifically). Unfortunately, research has not led to a conclusive determination as to whether or not GSH and its associated enzymes are increased or decreased in cases of Huntington’s Disease and in the overall disease mechanism within this particular focus due to conflicting results and thus hypotheses [94].

**Figure 2:**
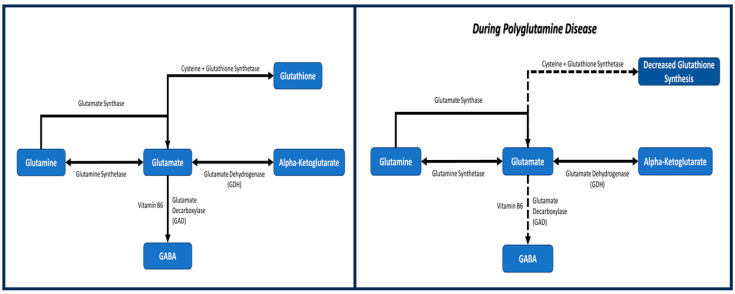
A distorted glutamine–glutamate cycle during polyglutamine diseases. A balance in the glutamine–glutamate cycle is very important for neuronal homeostasis. During polyglutamine diseases, this homeostasis is altered, resulting in either enhanced glutamate, reduced glutathione formation, or both.

Glutamine along with glutamate, alpha-ketoglutarate, GABA, and glutathione are all crucial components of energy metabolism and play roles in the development and progression of polyglutamine diseases. The glutamate–glutamine cycle, the citric acid cycle, the role of neurotransmitters, as well as the transporters involved with each of these molecules, can be studied to understand how dysfunctions arise in polyglutamine diseases. Glutamine regulation, including glutamate biosynthesis and glutathione generation, could be altered in polyglutamine diseases that contribute toward neuronal toxicity, as shown in Figure 2. Each of these metabolites provide researchers with multiple avenues for future therapeutics and treatments that are not limited to neurological components and considerations. By doing so, signals the importance and role of both biochemistry and energy metabolism in the realm of polyglutamine disease research.

## 6. Aberrant Nucleotide Biosynthesis in Polyglutamine Disease and Therapeutics

The presence of excess glutamine, particularly in those found in the polyglutamine tracts that cause polyglutamine diseases, could potentially have profound impacts on both nucleotide biosynthesis and regulation [95,96,97,98,99,100,101,102,103,104,105,106,107,108]. Purine and pyrimidine synthesis both require a number of glutamines to form correctly. Two glutamine molecules are required to generate inosine monophosphate (IMP), which acts as the predecessor to both purines, adenosine monophosphate and guanosine monophosphate (GMP) [109]. A third glutamine molecule is needed to assist the conversion of IMP to GMP. For pyrimidine synthesis, a single glutamine molecule is necessary for carbamoyl phosphate synthetase to produce uridine triphosphate (UTP) from ammonia and bicarbonate. UTP can then be used to produce cytidine triphosphate (CTP) with the use of a second glutamine molecule [95,96,97,98,99,100,101,102,103,104,105,106,107,108]. Outside of the de novo synthesis of nucleotides, glutamine assists and regulates nucleotide synthesis through a number of other methods. As previously mentioned, glutamine has a hand in the activation of mTORC1, which acts downstream to phosphorylate a complex of enzymes that produce orotate (a precursor of pyrimidines that helps form the pyrimidine ring) [95,96,97,98,99,100,101,102,103,104,105,106,107,108]. It also acts as an inducer for phosphoribosyl pyrophosphate amidotransferase (PPAT) by catalyzing the first step purine formation. Glutamine can also be converted into glutamate and glutathione, both of which have their own set of roles in metabolism and amino acid and nucleotide synthesis [95,96,97,98,99,100,101,102,103,104,105,106,107,108]. Any aberrations in these synthesis pathways due to excess glutamine production could result in various manifestations in DNA that manifest as symptoms of polyglutamine diseases.

### 6.1. Purine Metabolism and Polyglutamine Disease

The relationship between purine metabolism and the development of Huntington’s Disease has been more closely researched in recent years. As a group of nucleotides, purines act as metabolic signals within the body, helping to manage cell development and energy metabolism [110,111,112,113,114]. Thus, it is thought that impaired nucleotide metabolism in patients with Huntington’s Disease, more specifically purine synthesis and regulation, could cause extracellular aggregations of purines. Such aggregations could alter cellular signaling and act as an inducing factor for polyglutamine disease symptoms [110,111,112,113,114]. Research has found that the Huntingtin protein (Htt) increases cellular IMP levels, causing an acceleration of purine formation in the cell. This indicates that Htt has a role in purine balance and regulation [110,111,112,113,114]. In patients with Huntington’s Disease, decreased levels of hypoxanthine and increased levels of inosine and adenosine were noted [110,111,112,113,114]. Further, adenosine A1 receptors, i.e., those found in neurons, microglia, astrocytes, and oligodendrocytes, are dysfunctional in patients with Huntington’s Disease. This is significant because the normal function of A1 receptors are to minimize neuronal degradation by preventing excitatory transmissions from causing damage and regulating their expression. In addition, P2X7 and P2Y2 receptors hold crucial roles in Huntington’s Disease, with their signaling become impaired with the disease [110,111,112,113,114]. With Huntington’s Disease patients, a decrease in adenine nucleotides were found, likely due to a decrease in the energy metabolism of the cell. This decrease is thought to lead to increased nucleotide degradation and muscular atrophy, which are seen as symptoms in many polyglutamine diseases [110,111,112,113,114]. Mutant mice models have confirmed that the presence of Huntington’s Disease is correlated with an upregulation of adenosine deaminase transcription and enzyme activity, reduced adenosine levels, and increased inosine concentrations [115,116]. The lack of change with Purine Nucleoside Phosphorylase (the *Pnp* gene) and enzyme activity with an upregulation in transcription levels indicates that inosine degrades into hypoxanthine once outside skeletal muscle cells [115,116]. This hypoxanthine further degrades to xanthine and uric acid when aggregates pool together extracellularly. As a whole, this indicates an acceleration in purine synthesis [114]. While there are currently no clear findings in the literature about the role of purine synthesis and/or signaling with relation to Huntington’s Disease or other polyglutamine diseases, the research conducted thus far has reflected the start of further investigations into the possible relationships between polyglutamine diseases and purines, as well as the new and possible pathways for therapeutics against such diseases.

### 6.2. DNA Repair and Polyglutamine Disease

Another aspect of nucleotide synthesis involves DNA repair enzymes and pathways. In conditions where an abnormal number of nucleotides or the wrong type are produced, DNA repair pathways are activated to correct the imperfections in the genetic code [62,110,112,113,114,117,118,119,120]. Recently, researchers have hypothesized that these repair pathways regulate the onset of polyglutamine diseases, with longer CAG repeats that are uncorrected by DNA repair enzymes inducing an earlier age of onset in patients. Heritability is correlated to many of the polyglutamine diseases, prompting the partial activation of Huntington’s Disease, SCA2, and SCA3 [62,110,112,113,114,117,118,119,120]. The excess CAG repeats that code for glutamines cause the affected genes to be meiotically unstable, and this somatic instability affects the ability for DNA repair proteins to perform their roles properly, thus leading to cell death and physical symptoms of disease. A study conducted with just under 1500 people presenting with some type of confirmed polyglutamine disease found that each of the polyglutamine diseases underwent a shared pathway in which the genetic modifications within the DNA repair pathways specifically related to the age of onset of polyglutamine disease [62,110,112,113,114,117,118,119,120]. Further research into these patients allowed the researchers to consider that the somatic instability of the genes affected by polyglutamine disease are instead due to the formation of secondary structures of DNA that are not in their usual formation. It is thought that these unusual secondary structures caused by the excess glutamines can thus bind to DNA repair proteins that cause the instabilities in the genes. The most significant are those of a genetic variation that were found in rs3512 in the 3′ untranslated region of the *FAN1* gene, which has DNA endonuclease and exonuclease activity, thus proving that DNA repair pathways influence or become affected by the polyglutamine disease [19].

Nucleotides and the entire genetic code play an important role in the initiation and development of polyglutamine diseases. Due to the excess of glutamine repeats in a number of specific genes in this disease category, the regulation of glutamine through nucleotides is of the upmost importance as these nucleotides are necessary for glutamine to form. Excess glutamine and thus excess nucleotide formation can cause a number of abnormalities, including aggregates and cellular instability, which DNA repair pathways are not always able to address. Thus, targeting the synthesis of nucleotides, the DNA repair mechanisms to repair incorrectly, or excess nucleotides, as well as addressing the prevention of aggregation, are all areas in which further understanding about the role of nucleotides can be useful in developing treatments for those with polyglutamine disease.

## 7. Endogenous Antioxidant Regulation and Protective Mechanisms during Polyglutamine Disease

Oxidative stress has emerged as an important underlier for the potential progression of polyglutamine diseases, as well as for avenues of treatment. To maintain proper function, cells attempt to utilize oxidants and antioxidants in their cellular system. When this regulation is imbalanced, oxidative stress occurs, usually in the form of larger than normal amounts of reactive oxygen species errors in the signaling pathways or impaired mitochondrial or cellular functioning [121,122,123,124,125,126,127]. Reactive oxygen species’ (ROS) activity in particular has proven to be an important consideration as it can be reduced by the use of antioxidants (both enzymatic and non-enzymatic ones). While enzymatic antioxidants are those that convert the ROS into less toxic compounds, non-enzymatic antioxidants act by scavenging free radical species, inhibiting ROS formation, and inducing further antioxidant production in the cell [109]. Current research on the relationship has found that polyQ stretches are associated with not only oxidative stress, but also associated proteasome impairments and microglial activation that can further exacerbate oxidative stress and its negative effects [121,122,123,124,125,126,127].

Neurodegeneration and oxidative stress processes are intertwined in many ways. Research looking into the relationships between the two have most commonly been conducted using induced pluripotent stem cells (iPSCs) and animal models [128,129,130]. Patients with SCA3, for example, have been found to have higher than normal levels of ROS in their serum, while the brains of patients with Huntington’s Disease have been found to have higher amounts of oxidative damage alongside the lessening of mitochondrial DNA and potential dysfunction [28]. Experimentation has found that SCA3 patients with long polyQ repeats and increased glutamines have an abnormal reduced:oxidized glutathione ratio, which is indicative of oxidative stress [128,129,130]. Furthermore, research with polyQ-inflicted rats have confirmed the generation of ROS and the decreased activity of enzymatic antioxidants, as well as DNA damage, and these results are similar to those found in the human fibroblasts of patients with Huntington’s Disease and SCA2 [121,124,131,132,133]. SCA1 patients have presented with an accumulation of ROS products and subsequent degradation of oxidative phosphorylation pathways have been shown to cause ATP synthesis deficiency [121]. Each of these experiments have supplied new insight into the existence and role of oxidative stress, but it is still unclear as to how exactly the oxidative burden is increased by extended polyglutamine stretches. Current hypotheses revolve around the idea that the polyQ proteins cause an increased production in ROS that induce the oxidative stress in the cellular system. For Huntington’s Disease, excess glutamine repeats in the promoter region of the Htt gene in mice. Furthermore, there is a correlation in the length and severity in addition to the increased glutathione levels and reduced complex II and IV activity, all of which are thought to be related to an increased oxidative burden [128,129,130]. SCA1 was found to also have changes in its mitochondrial protein levels and expression, which is thought to be related to elevated oxidative stress [121]. The proteasome and its ability to tag items to be degraded with ubiquitin has impaired function in Huntington’s Disease patients’ brains, thus contributing toward pathogenesis.

### 7.1. Role of Autophagy in Polyglutamine Disease

Autophagy also holds a big role in the regulation of oxidative stressors in polyglutamine disease. The polyQ stretches in an affected Huntington’s Disease patients are thought to prevent Htt relocation, increasing the number of autophagic vesicles. The presence of mutant Htt impairs the cell’s ability to degrade its components, and this can cause defects in cargo loading. Selective degradation of the mitochondria through autophagy pathways, also known as mitophagy, has been determined to be suppressed in polyglutamine conditions. In Huntington’s Disease, for example, the Htt acts as a framework, holding protein complexes in place and interacting with signaling molecules and receptors to induce the degradation of damaged mitochondria in need of autophagy. PolyQ conditions create dysfunction in these processes, thus impairing the mitophagy [122].

### 7.2. Neuroinflammation Mediated Exacerbation of Pathological State

Neuroinflammation is another important consideration in the relationship between oxidative stress and neurodegenerative diseases such as the group of polyQ diseases. Glial cells as a whole are the usual mediators of neuroinflammation and can take pro-inflammatory or anti-inflammatory roles [134,135,136,137,138,139]. Furthermore, activated glial cells can produce both reactive oxygen and nitrogen species, thus adding to the oxidative stress that is already experienced by neurons [134,135,136,137,138,139]. Huntington’s Disease results in the production of a mutant HTT protein that causes an increased presence of pro-inflammatory cytokines that are able to induce caspases and the production of free radicals. This causes a migration of immune cells to the brain, leading to neuroinflammation and exacerbating symptoms [140]. Furthermore, the presence of mutant *HTT* relates to imbalances in the synaptic and extra-synaptic signaling. This is partially due to neuronal death causing switches in microglial states (from the surveillance state and with microglia functioning as chaperones in the neurons) to the activated state (with the microglia causing neuroinflammation) [141]. SCA1 patients have been found to undergo similar immune-mediated neurodegeneration due to neuroinflammation [140].

### 7.3. Endogenous Antioxidants in Combating Cellular Damage

Specific antioxidants and other similar molecules provide different effects toward the endogenous regulation of the oxidative stressors within cells, and they could serve as novel therapeutics. In SCA3 patients, for example, the administration of creatine was seen to improve motor performance along with mitochondrial function, thus indicating that creatine may hold a beneficial role in preventing the further progression of SCA3 pathology and progression in patients [142,143]. A number of antioxidants have been tested for possible benefits against Huntington’s Disease, including vitamin C, selenium, fatty acids, etc. Vitamin C has been found to counteract glutamate-induced neurodegeneration, as well as respond to cells in a general restorative manner [144,145,146]. Selenium can act in a number of pathways, particularly within neurotransmissions, but it also shows neuroprotective activity like other antioxidants [141]. Unsaturated fatty acids have also been shown to hold protective effects against oxidative damage, thus leading the way in terms of supporting the importance of diet and the supplementation of antioxidants in efforts to achieve oxidative protection (especially for patients with polyglutamine disease diagnoses) [147]. Often activated by the alterations of neuronal activity, reactive microglia have been known to have an important presence in Huntington’s Disease patients and, presumably, all other SCAs and polyglutamine diseases [148]. The extent of reactive microglial presence has been linked to the severity of the disease and often occurs alongside proteasome activation and in the excess production of free radicals [149]. Antioxidants and free radical scavengers can be used to provide neuroprotective effects and minimize oxidative damage, thus alleviating the proliferation of polyglutamine disease. Modifying existing endogenous antioxidant pathways, especially in glia and microglial cells, in addition to lifestyle modifications such as dietary changes could act as promising therapeutic strategies for polyglutamine and, more broadly, neurodegenerative diseases.

## 8. Novel Therapies for Polyglutamine Diseases

Novel therapies have taken a multitude of approaches to treating and preventing the further development of polyglutamine diseases. In the last ten years particularly, nanomedicine-based therapies, neurorehabilitation, cerebellar stimulations, as well as numerous drugs, have been developed, introduced, and shown to counteract the development of polyglutamine diseases through a variation of biochemical and metabolic mechanisms.

Nanomedicines have become a significant focus within polyglutamine disease therapeutics. Drug discovery has focused on creating nanoparticles that can target cell surface receptors and aid with the encapsulation of therapeutics in order to penetrate the blood–brain barrier and prevent their easy degradation. For the treatment of polyglutamine diseases specifically, the most common nanoparticles used include liposomes and solid lipid nanoparticles in various doses via intracranial, intranasal, oral, intravenous, and intraperitoneal routes of administration [150]. As of now, these polyglutamine-disease-targeting nanomedicines either utilize nucleic acids, small peptides, or small molecules. Nucleic acid-based therapies could directly target the fragments of DNA that actually cause the disease. Researchers have found that the siRNA-loaded nanopeptides complexed to target genes play a major role in the pathogenesis of polyglutamine diseases. Small peptides are currently being used as peptide inhibitors, and they are designed to de-aggregate proteins that have already been misfolded due to polyglutamine disease. When loaded with different peptide inhibitors, nanopeptides were found to protect against proteolytic degradation and assist with delivery to targets in the body, and those who were administered these peptides had better motor performance than those who were not [151]. In addition, the injection of a nano-Vascular Endothelial Growth Factor (VEGF), a small peptide that is often decreased in those with SCA1, was found to improve the latency to fall and increased walking speeds of mice who had received such injections for a span of two weeks [124]. These findings offer some promises for nanoparticle- and nanomedicine-based therapy for polyglutamine diseases.

Furthermore, a number of clinical studies have been conducted to evaluate the effectiveness of numerous therapies and procedures to alleviate symptoms of polyglutamine diseases. A clinical trial explored the effects of neurorehabilitation therapy in SCA2 patients. Participants were enrolled in a 24-week course, in which they received 6 h of rehabilitation each weekday, including an hour of occupational therapy, a half hour of general conditioning, four hours of physical therapy, and a half hour of psychotherapy. The research team found that those who engaged in the 24 weeks of therapy had a reduction in ataxia severity, as shown by the decrease in the Scale for Assessment and Rating of Ataxia (SARA) score compared to those in the control group. Treatment group patients also had improvements in their cerebellar signs, ataxic gait, postural stability, and limb dysmetrias. One caveat of these results, however, was that those who were older and/or with shorter CAG repeats experienced the most drastic reduction in SARA scoring [152].

Similar to the previously mentioned work, another clinical trial that took place in the Netherlands looked into the effects of cerebellar anodal transcranial direct current stimulation (tDCS) in patients with SCA3. Patients were subject to a two-week treatment regimen composed of ten sessions of daily cerebellar stimulation, which was found to alleviate ataxia and non-motor system severity in those with SCA3. Researchers noted that the SARA scores in the patients who underwent the treatment regimen did not have any statistically different changes compared to that of the control group; however, select patients did have a sustained reduction in their SARA scoring over the span of six to twelve months, thus leading clinicians to believe that there is interindividual variability in the effectiveness of this type of therapy. These results indicate that, although not for everyone, cerebellar anodal transcranial direct current stimulation should be kept as an option for polyglutamine disease patients for cases where their condition and the manifestation of the disease makes them a good candidate for treatment through this modality [153].

Furthermore, investigations into the targeting of protein misfolding machinery have proven to be a new angle for developing treatment. Antioxidants, as mentioned above, are another realm of therapies that are being explored for the treatment of polyglutamine diseases. Tolfenamic acid, a drug with high antioxidant activity, has been found to improve motor coordination and memory in patients [154]. In a similar manner, another study showed that N-acetylcysteine [58] delays the onset and progression of motor defects in those it is administered to, and it was also found to rescue the mitochondrial respiratory deficits in the brain along with levels of carbonylated proteins [154]. Clinical trials have already begun to explore the role of antioxidants in analogous ways, with d-alpha-tocopherol (vitamin E) acting as a beneficial addition toward disease prevention and regression when administered early in the disease progression of Huntington’s Disease patients [155]. Additionally, the use of highly unsaturated fatty acids (HUFAs) and the ethyl-ester of eicosapentaenoic acid (ethyl-EPA) as part of a treatment for Huntington’s Disease patients was found to produce significant motor improvements in patients [156,157].

Current research into polyglutamine disease treatment modalities has included a variety of upregulations in the multiple enzymes and transporters that are crucial for the correction of mutant gene formations. The upregulation of Sirt1, an enzyme known to protect motor neurons, for example, has been found to be beneficial in reducing polyglutamine disease symptoms in patients with Huntington’s Disease [82]. Similarly, the upregulation or the addition of proteasomal activators has been found to be beneficial for polyglutamine disease treatment [140]. Benzamil and baclofen are two drugs that activate the proteasome, and they have also been found to improve motor coordination and limit the overall motor deficits in patients [158]. Beta lactam antibiotics have been found to stimulate GLT-1 expression and, correspondingly, glutamate uptake through the increased transcription of GLT-1 [159]. Also, increasing intracellular and extracellular adenosine levels is thought to be helpful as treatment as adenosine is a crucial neuronal and protein degradation regulator [114].

Numerous drugs have been proposed for use to treat polyglutamine diseases, particularly Huntington’s Disease. Overall, many of these compounds enhance inhibitory GABAergic transmissions of neurons through receptor targeting and the production of muscle relaxing effects [92]. Alprazolam is a benzodiazepine-activating GABA receptor that was found to reverse dysregulated circadian rhythms and improve the cognitive performance of mice with Huntington’s Disease [160]. Progesterone is a positive regulator of GABA receptors, and its overexpression significantly reverses behavioral impairments in subjects [161]. Other classes of drugs can also adjust the concentration of GABA within the synapse to address polyglutamine disease symptoms. Tiagabine is a medication used to block the GABA transporter GAT1 in order to increase synaptic GABA concentrations [162]. Mice treated with this agent have improved motor performance and survival times compared to those who were not [162]. Vigabatrin is a GABA-T inhibitor that blocks GABA catabolism in both neurons and astrocytes, and it has also been found to have positive effects toward alleviating symptoms [163]. The use of taurine as a drug has been found to not only increase GABA levels, but also improve locomotor defects because of polyglutamine disease [164]. All these pharmacological agents have contributed toward alleviating polyglutamine disease symptoms.

The advancement of research into polyglutamine disease formation, development, and progression has led to an enormous spread of new and novel therapies and treatments. Knowledge about the metabolic and biochemical pathways that govern disease initiation has supplied avenues of treatment that are not only limited to supportive care or prevention, but also the targeting of enzymes, protein folding modifiers, transporters, antioxidants, molecules, and even nucleic acid changes that can be administered to patients in an equally wide array of methods. While pharmaceutical agents including nanomedicines are common modes of treatment, non-pharmacological strategies like rehabilitative therapy have also demonstrated promising results.

## 9. Conclusions

This review has focused on the current research and perspectives regarding polyglutamine diseases; this was achieved by looking at how they develop, progress in severity, and how they can be treated, all from a metabolic angle. Although many of these gatherings are not knew in the research world, seeing these topics from a metabolic angle will certainly be of note as more biological processes are understood within the context of polyglutamine diseases and, correspondingly, more treatments are explored and created to address the core causes of the disease along with its related clinical symptoms. Looking to the future, the metabolic components addressed here also could also be useful in the diagnosis and treatment of polyglutamine diseases, and it should be used as such. With this group of diseases in particular, nanomedicines are seen to be the method of the future for drug delivery and efficacy, and this will be followed by several pharmaceuticals, many of which will utilize antioxidants and other naturally occurring bodily molecules that address the enzymatic or biological process-based components of disease progression. For these reasons, continued research into polyglutamine diseases and their therapeutics are of utmost importance in providing a better quality of life for those who inherit polyglutamine diseases.

## Figures and Tables

**Table 1:** List of polyglutamine diseases, their major clinical features, and the genes involved.

PolyQ Disease	Gene	Major Clinical Features	Source
Dentatorubropallidoluysian Altrophy (DRPLA)	*ATN1*	Ataxia and progressive myoclonus epilepsy (childhood onset), repeated seizures and potential myoclonus (early adult onset), ataxia, choreoathetosis, and dementia (late adult onset)	Smith, 1958 [48]
Huntington’s Disease (HD)	*HTT*	Progressive chorea and cognitive and behavioral symptoms	Ghosh, 2018 [11]
Spinal and Bulbar Muscular Atrophy (SBMA)/Kennedy’s Disease	*AR* (X-linked)	Postural tremors and muscle cramps, followed by progressive muscle weakness, which have distinguishing features that include profound facial fasciculations, bulbar signs, gynecomastia, and sensory disturbances	Atsuta, 2006 [14]
Spinocerebellar Ataxia Type 1 (SCA1)	*ATXN1*	Progressive ataxia, impaired cognition, weakening of eye muscles, dysarthria, and dysphagia	Sasaki, 1996 [13]
Spinocerebellar Ataxia Type 2 (SCA2)	*ATXN2*	Progressive ataxia, dysarthria, postural tremors, slow saccades, and hyporeflexia	Cancel, 1997 [27]
Spinocerebellar Ataxia Type 3 (SCA3)/Machado–Joseph Disease (MJD)	*ATXN3*	Progressive cerebellar ataxia, areflexia, spasticity, and muscle atrophy	Sequeiros, 1993 [49]
Spinocerebellar Ataxia Type 6 (SCA6)	*CACNA1A*	Progressive ataxia, dysarthria, dysphagia, intention tremors, positional vertigo, nystagmus, and other motor and muscular defects	Stevanin, 1997 [50]
Spinocerebellar Ataxia Type 7 (SCA7)	*ATXN7*	Macular and/or retinal degeneration with vision loss, slow saccades, ophthalmoplegia, progressive ataxia, dysphagia, and respiratory distress	David, 1997 [42]
Spinocerebellar Ataxia Type 17 (SCA17)	*TBP*	Progressive gait and limb ataxia; seizures; neurologic, cognitive, and/or psychiatric impairments; and pyramidal and extrapyramidal features such as spasticity, chorea, and dystonia	Koide, 1999 [43]

## Data Availability

Not applicable.
